# The Role of Health Literacy among Outpatient Caregivers during the COVID-19 Pandemic

**DOI:** 10.3390/ijerph182211743

**Published:** 2021-11-09

**Authors:** Elisabeth Rohwer, Natascha Mojtahedzadeh, Felix Alexander Neumann, Albert Nienhaus, Matthias Augustin, Volker Harth, Birgit-Christiane Zyriax, Stefanie Mache

**Affiliations:** 1 Institute for Occupational and Maritime Medicine (ZfAM), University Medical Center Hamburg-Eppendorf (UKE), Seewartenstr. 10, Hs. 1, 20459 Hamburg, Germany; n.mojtahedzadeh@uke.de (N.M.); harth@uke.de (V.H.); s.mache@uke.de (S.M.); 2 Midwifery Science-Health Services Research and Prevention, Institute for Health Service Research in Dermatology and Nursing (IVDP), University Medical Center Hamburg-Eppendorf (UKE), Martinistr. 52, 20246 Hamburg, Germany; Fe.Neumann@uke.de (F.A.N.); b.zyriax@uke.de (B.-C.Z.); 3 Department of Occupational Medicine, Hazardous Substances and Public Health, Institution for Statutory Accident Insurance and Prevention in the Health and Welfare Services (BGW), Pappelallee 33/35/37, 22089 Hamburg, Germany; a.nienhaus@uke.de; 4 Institute for Health Service Research in Dermatology and Nursing (IVDP), Competence Centre for Epidemiology and Health Services Research for Healthcare Professionals (CVcare), University Medical Centre Hamburg-Eppendorf (UKE), Martinistr. 52, 20246 Hamburg, Germany; 5 Institute for Health Service Research in Dermatology and Nursing (IVDP), Competence Centre for Health Services Research in Vascular Diseases (CVvasc), University Medical Center Hamburg-Eppendorf (UKE), Martinistr. 52, 20246 Hamburg, Germany; m.augustin@uke.de

**Keywords:** outpatient care, ambulatory care, health behaviour, nutrition, physical activity, stress, worries, information sufficiency, coronavirus, pandemic

## Abstract

Health literacy became an important competence during the COVID-19 pandemic. Despite outpatient caregivers being a particularly vulnerable occupational group, their health literacy has hardly been examined yet, especially during the pandemic. Hence, this study aimed to explore this field and provide first empirical insights. Data were collected based on a cross-sectional online survey among 155 outpatient caregivers. In particular, health literacy (HLS-EU-Q16), diet and physical activity, pandemic-related worries, perceived information sufficiency and stress perception were examined. Descriptive and ordinal logistic regression analyses were run to test explorative assumptions. The majority of outpatient caregivers reported high values of health literacy (69% on a sufficient level). Although no significant associations between health literacy and health behaviours or perceived information sufficiency were found, perceived information sufficiency and perceived stress (*OR* = 3.194; 95% CI: 1.542–6.614), and pandemic-related worries (*OR* = 3.073; 95% CI: 1.471–6.421; *OR* = 4.243; 95% CI: 2.027–8.884) seem to be related. Therefore, dissemination of reliable information and resource-building measures to reduce worries may be important parameters for improving outpatient caregivers’ health. Our results provide first explorative insights, representing a starting point for further research. Considering outpatient caregivers’ mobile work setting, they need to be provided with adequate equipment and comprehensible information to ensure physically and mentally healthy working conditions.

## 1. Introduction

The Coronavirus Disease 2019 (COVID-19) pandemic as a global health problem highlights the importance of health literacy. Health literacy shapes health behaviours of individuals. During the ongoing pandemic situation, health behaviours, among numerous aspects of everyday life, have changed to prevent infection with the coronavirus [1,2]. Besides the risk of contracting SARS-CoV-2, restrictive regulations leading to these behavioural changes, as well as misinformation about the pandemic, may impact individuals’ (mental) health [3,4] by causing panic and anxiety [5]. The possibility of obtaining information through different media may lead to an ‘infodemic’, making it difficult for individuals to identify trustworthy and reliable information [6]. Therefore, sufficient health literacy as a key factor in improving health and well-being [2] should be considered a vital component in dealing with the psychological consequences of the pandemic [5].

Although health literacy has often been researched in the context of patients with chronic diseases (e.g., hypertension, asthma, cancer, or HIV/AIDS) [7,8,9]), it has rarely been examined among outpatient caregivers in scientific studies so far [10]. Before the COVID-19 pandemic, in 2019, the occupational group of outpatient caregivers in Germany consisted of 421,550 employees, most of whom were trained as geriatric nurses (98,976) or geriatric care assistants (21,831) and as health and care nurses (78,129) or nursing assistants (14,822). In Germany, 80% (3.3 million) out of 4.1 million persons in need for care were cared for at home at that time. With an ongoing demographic change in Germany, and thus a greater need for (geriatric) care, their job is highly relevant to society and the number of outpatient care services is increasing [11]. Outpatient caregivers typically work in a mobile setting, while their work activities include body-related care measures, nursing care measures, and home nursing. These include, for example, helping with personal hygiene, nutrition, promotion of mobility and orientation, organising everyday life and administering medication or changing bandages [12].

### 1.1. Health Literacy as a Crucial Factor for Health

The term health literacy refers to both the individual and public health level, and is linked to literacy in general. Based on an integrative review on health literacy definitions and models, Sørensen, et al. [13] stated that health literacy “entails people’s knowledge, motivation and competences to access, understand, appraise, and apply health information in order to make judgments and take decisions in everyday life concerning healthcare, disease prevention and health promotion to maintain or improve quality of life during the life course” [13]. 

### 1.2. Health Literacy among Healthcare Workers

A German exploratory interview study among nursing staff in hospitals indicated that the subjective health literacy, more specifically exhibiting beneficial health behaviours, is rather limited due to specific job demands such as lack of staff and time pressure [14]. In the period before the COVID-19 pandemic, a German study among outpatient nurses for the elderly, almost 40% of the participants reported difficulties in dealing with health-related information (0–8 points on the 16-point HLS-EU-Q16). Thirteen percent even showed problematic health literacy (9–12 points), whereas 49% showed sufficient health literacy (13–16 points). Respondents with difficulties in handling health-related information have a 13.48-fold increased odds of having frequent thoughts of changing careers [10]. With regard to the COVID-19 pandemic, understanding specific health- and protection-related information has become even more important. The importance of information transmission has already been described as an important occupational health and safety measure in a recent interview study among outpatient caregivers form Germany [15]. However, it remains unclear how health literacy and perceived information sufficiency are related. Therefore, we explore the following assumption:

**Assumption** **1:** *The outpatient caregivers’ health literacy is significantly associated with their perceived sufficiency of information.*

### 1.3. Health Literacy and Health Behaviour

Research by Jordan and Hoebel [16] provides support for the assumption that health literacy and health behaviour are associated. A relation was found between inadequate health literacy and a lower prevalence for physical and sporting activity as well as for fruit and vegetable consumption [16]. In a study of British adults, higher health literacy led to increased fruit and vegetable consumption per day and increased the likelihood of being a non-smoker and having good subjectively rated health [17]. A relationship between health literacy and physical activity could moreover be partially supported in a previous study among working adults from Germany [18].

### 1.4. Health Behaviour among Healthcare Workers

Coping with work-related stress through unhealthy eating, such as sweets, was further supported by a recent German interview study among 15 outpatient caregivers. Likewise, work-related exhaustion was reported as a reason for lower physical activity during leisure. Nevertheless, participants also described a high amount of work-related physical activity as stressful, which could be another explanation for reduced physical activity during leisure [19]. However, a study among Korean hospital nurses did not find significant relationships of eHealth literacy with physical activity and nutrition. The authors justify this finding by indicating that the gap between the nurses’ well-developed health and their actual health behaviours may be related to specific job demands such as time pressure and limited access to exercise facilities and healthy food choices [20]. Among Vietnamese undergraduate nursing students, both health literacy and higher scores of a newly developed scale for digital healthy diet literacy, were associated with a higher likelihood of healthier eating behaviour during the COVID-19 pandemic, respectively [21]. Nurses in a US hospital exhibited a more irregular meal pattern when they were more stressed. Most frequently, approaches applied to coping with stress among the sample included eating, exercising, relaxation techniques (e.g., yoga, meditation), and talking with family members or friends [22]. The influence of shift work on nurses’ diet was investigated in an Australian study: higher levels of stress were also related to a higher energy intake as well as a higher percentage of fat and saturated fat intake [23]. Based on these findings, we assume a relationship between health literacy and health behaviours among outpatient caregivers.

**Assumption** **2a:** *The outpatient caregivers’ health literacy is significantly associated with their diet.*

**Assumption** **2b:** *The outpatient caregivers’ health literacy is significantly associated with their physical activity.*

### 1.5. Health Literacy, Perceived Information Sufficiency and Pandemic-Related Worries

Despite demonstrated associations of health literacy and beneficial health behaviours, there is some evidence that higher levels of health literacy might even lead to maladaptive health behaviours in times of anxiety. Therefore, depending on the circumstances, health literacy may also have adverse effects for anxious individuals [24,25]. Among US adults, concerns about economic consequences of the COVID-19 pandemic, health-related worries and social distancing were related to poorer mental health worries [26]. A study among the general Norwegian population revealed an association of substantial health-related worries and psychological distress with emotional eating, respectively. Women and participants in their 30 s were more inclined to emotional eating [27]. Moreover, a cross-sectional study from Germany indicates that despite high levels of health literacy among participants, dealing with pandemic-related information can be challenging, nevertheless. Furthermore, lower levels of health literacy were associated with more confusion about pandemic-related information [28]. Therefore, further research is needed to examine whether health literacy might be related to perceived information sufficiency.

### 1.6. Worries during the COVID-19 Pandemic among Healthcare Workers

In a Vietnamese study among healthcare workers from hospitals and healthcare centres from April 2020, healthcare workers involved in COVID-19 response with low health literacy had a 12.06 times higher likelihood of anxiety, 7.84 times higher depression, and 5 times lower health-related quality of life scores, whereas healthcare workers uninvolved in COVID-19 response with higher health literacy had a 45% lower likelihood of anxiety and 42% lower depression and 2.53 times higher health-related quality of life scores. When they were more health literate, healthcare workers who were involved in the COVID-19 response had a 43% lower likelihood of anxiety and 37% lower depression as well as higher health-related quality of life scores (1.10 times higher) [29]. A high degree of pandemic-related worries was found in a sample of UK hospital trainees; 91.6% were worried about COVID-19, mostly due to the risk of family members or friends dying from (70.4%) or being infected with (67.6%) the disease, as well as transmitting it to them after an infection at work (59.8%). In contrast, the fear of infecting themselves as while working in the care sector was comparably lower (27.4%). Perceived information sufficiency about the COVID-19 pandemic was high (50–80%) [30]. However, a US study did not reveal differences in patterns of COVID-19-related worries, anxiety and depression between healthcare providers and non-healthcare providers. The results suggest that healthcare providers, regardless of their profession, are concerned about the pandemic [31]. Several Chinese studies within the context of the COVID-19 pandemic among healthcare workers found that almost half of the participants showed symptoms of anxiety [32,33]. Moreover, frontline healthcare workers had a higher risk of anxiety and distress [32]. Proceeding from these findings, we explore the following assumption:

**Assumption** **3:** *The outpatient caregivers’ health literacy is significantly associated with their pandemic-related worries.*

Although there are no studies among healthcare workers yet, several scientific studies emphasise the relation between stress perception and worry due to the COVID-19 pandemic with eating behaviour among the Norwegian population [27]. Pandemic-related worries were associated with increased food intake, which had emotional motives. This also led to psychological stress which was associated with emotional eating behaviour, as well [27]. This led us to explore the following assumptions presented below:

**Assumption** **4a:** *The outpatient caregivers’ pandemic-related worries are significantly associated with their diet.*

**Assumption** **4b:** *The outpatient caregivers’ pandemic-related worries are significantly associated with their physical activity.*

### 1.7. Perceived Information Sufficiency and Perceived Stress during the COVID-19 Pandemic among Healthcare Workers

According to an Arabic study, most healthcare workers obtain their information on COVID-19 via social media. The sample of healthcare workers mostly had poor knowledge of SARS-CoV-2 transmission (61.0%) and symptom onset (63.6%) in the beginning of the COVID-19 pandemic [34]. Among Saudi-Arabian healthcare workers, infection control attitudes were positively correlated with their perceived adequacy of information on the COVID-19 pandemic. Hygienic practice was further correlated with perceived adequacy of information and participants’ knowledge. Moreover, having participated in an education day by the hospital reduced the likelihood of high stress perception during COVID-19 compared to MERS-CoV [35]. Findings from Germany indicate that caregiving relatives felt they were sufficiently informed about the ongoing pandemic situation, although they were emotionally stressed and exhausted [36]. Among Swedish ambulance nurses, perceiving information insufficiency led to higher levels of stress perception [37], whereas perceived uncertainty and unfamiliarity in the early stages of the coronavirus outbreak led to exhaustion among Chinese healthcare workers [38]. We therefore pursue the following assumption:

**Assumption** **5:** *The outpatient caregivers’ perceived sufficiency of information is significantly associated with their level of perceived stress.*

Interestingly, Saudi-Arabian healthcare workers who were previously exposed to cases of MERS-CoV were less likely to perceive high levels of stress during the COVID-19 pandemic. Still, their worries of contracting COVID-19 were associated with high levels of stress compared to the MERS-CoV pandemic a few years earlier. Moreover, their level of agreement with infection control measures was positively associated with higher stress levels during the COVID-19 pandemic. The findings indicate that previous pandemic experiences and awareness campaigns support knowledge improvement and may lead to reduced anxiety among healthcare workers during a pandemic in general [35]. A recently published interview study among outpatient caregivers reveals that German outpatient caregivers already perceived stress at work on a daily basis even before the COVID-19 pandemic [19]. Further study results show that job demands increased during the COVID-19 pandemic, and thus, stress perception, as a negative strain reaction of outpatient caregivers, also increased [39]. A current mixed-methods study from the COVID-19 context even provides insights of higher levels of perceived stress among outpatient caregivers during the pandemic [40]. In a study among hospital staff within the context of the A/H1N1 influenza pandemic, participants’ perceived information sufficiency was significantly associated with their degree of worry about the pandemic. Degree of pandemic-related worry, in turn, and to a certain extent perceived sufficiency of information, as well, were significantly related to the hospital staff’s general psychological distress [41]. Drawing upon these findings from the current and another pandemic situation, we assume that:

**Assumption** **6:** *The outpatient caregivers’ perceived sufficiency of information is significantly associated with their pandemic-related worries.*

**Assumption** **7:** *The outpatient caregivers’ pandemic-related worries are significantly associated with their level of perceived stress.*

### 1.8. The Moderating Role of Pandemic-Related Worries

In the context of the current COVID-19 pandemic, a US study found that participants were more reactive to stress when they worried more about the pandemic. Conversely, the buffering effect positive affect had on their stress levels was amplified when participants were less worried about the pandemic [42]. Based on these initial findings of the moderating role of pandemic-related worries, we propose the following assumptions for explorative investigations:

**Assumption** **8a:** *Outpatient caregivers’ pandemic-related worries significantly moderate the association between their health literacy and eating behaviour.*

**Assumption** **8b:** *Outpatient caregivers’ pandemic-related worries significantly moderate the association between their health literacy and physical activity.*

**Assumption** **9:** *Outpatient caregivers’ pandemic-related worries significantly moderate the association between their perceived sufficiency of information and their perceived stress.*

**Figure 1:**
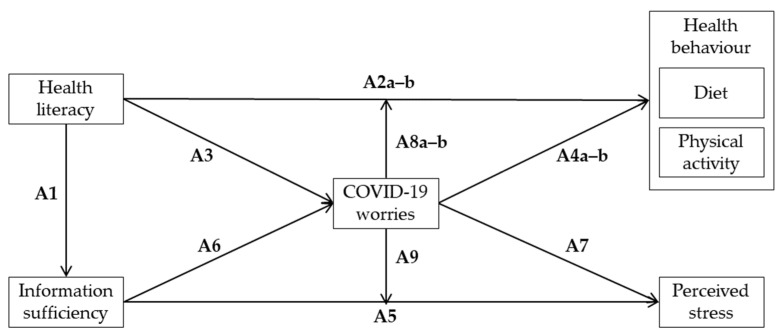
Overview of exploratory assumptions (own depiction).

Figure 1 provides an overview of our assumptions:

### 1.9. Study Aims

Our study aims to investigate the influential role health literacy played for outpatient caregivers’ health during the first year of the COVID-19 pandemic in Germany. Therefore, we examine the relationships of health literacy with health behaviour, perceived information sufficiency regarding the COVID-19 pandemic as well as pandemic-related worries, and perceived stress among outpatient caregivers in Germany. In this vein, we aim to provide empirical evidence on health literacy among outpatient caregivers and to contribute to filling the aforementioned research gaps.

## 2. Materials and Methods

### 2.1. Study Design and Recruitment of Participants

The study was designed as a cross-sectional online survey for outpatient caregivers in Northern Germany. Data were collected between May 2020 and February 2021. Inclusion criteria for study participation were (1) working as an outpatient caregiver (2) with the same employer for at least six months (3) with a workload of at least 15 h per week. Participants were recruited in multiple ways: a total of 367 ambulatory care companies were contacted by telephone. Additionally, the study information was distributed via email distribution lists and through groups of outpatient caregivers on social media (Facebook, Xing). The online survey homepage was visited 607 times. The survey was completed by *n* = 171 participants (28.2% of homepage visitors), whereas *n* = 315 (51.9%) dropped out and *n* = 121 (19.9%) did not actually participate in the survey. A total of 16 participants were excluded from data analyses due to missing or implausible values, resulting in a final sample size of *N* = 155.

### 2.2. Variables and Measurements

#### 2.2.1. Sociodemographic and Workplace Variables

To gather sociodemographic information on the participants, self-constructed items were used for gender, age, postcode and district as well as work area of outpatient care in which participants worked, general work experience and work experience in the current company, managerial responsibility, nationality, type of employment, full or part-time work, shift work, height, body weight, marital status, number of children, country of birth, mother tongue, and highest level of education. The body mass index (BMI) was calculated from participants’ height and body weight (BMI = (body weight in kg)/(body height in m)^2^), with overweight defined as a BMI  ≥  25 kg/m^2^ and obesity as BMI  ≥  30 kg/m^2^, according to WHO classification [43].

#### 2.2.2. Health Literacy

Health literacy was assessed using the instrument developed within the framework of the European Health Literacy Survey (HLS-EU-Q) [44] in its 16-item version (HLS-EU-Q16). This short form of the HLS-EU-Q47 measures health literacy within the three domains of healthcare, disease prevention, and health promotion. Across these three domains, the items address how easily participants can access, understand, appraise, and apply provided health information, e.g., “On a scale from very easy to very difficult, how easy would you say it is to judge if the information about illness in the media is reliable?”. The 16 items of this short version are internally consistent (Cronbach’s α = 0.90) and represent the 47-item long form (*r* = 0.82, *p* < 0.01) [16,45]. Thus, the German version was found reliable and valid [45,46]. The items were scored on a 4-point Likert scale (1 = *very easy*, 2 = *easy*, 3 = *difficult*, 4 = *very difficult*) and dichotomised (1 and 2 into 1 and 3 and 4 into 0) to create an individual sum score, ranging from zero (lowest health literacy) to 16 (maximum health literacy). This score was then divided into three categories: *inadequate* (0–8 points), *problematic* (9–12 points) and *sufficient* (13–16 points) health literacy [45].

#### 2.2.3. Health Behaviours

Eating Behaviour

Eating behaviour was measured using the German version of the Mediterranean Diet Adherence Screener (MEDAS) [47]. This instrument offers the advantage of assessing the overall dietary quality instead of only measuring individual items. Its 14-item scale surveys the consumption frequency of foods (olive oil, vegetables, fruit, red meat, animal fats, carbonated beverages, red wine per day and fish/seafood, legumes, nuts, commercial food, and Mediterranean traditional dishes with tomato sauce per week) with 12 items on a six-point scale (e.g., “How many pieces of fruit (including fresh-squeezed juice) do you consume per day?” on a scale from 1 = *less than once*, 2 = *once*, 3 = *twice*, 4 = *three times*, 5 = *four times* to *6 = five times a day or more* or, respectively, “How many times do you consume nuts per week?” on a scale from 1 = *less than once*, 2 = *once*, 3 = *twice*, 4 = *three times*, 5 = *four times* to 6 = *five times a week or more*). Additionally, two dichotomous items measured the preferred cooking fat used and meat consumed (*yes* or *no*). This diet is associated with lower risks of cardiovascular diseases and mortality [48,49] and has already resulted in positive effects in interventional trials [50,51]. A final score ranging from 0–14 was computed by scoring the items with zero (for *no*) or one (for *yes*) based on the suggestions by Schröder et al. (2011) [52] and Hebestreit et al. (2017) [47]. According to Schröder et al. (2011), the scale can be considered valid (*r* = 0.52, *p* < 0.001) [52]. More recently, Hebestreit et al. (2017) successfully validated the German version by comparison to the well-established Food Frequency Questionnaire [47].

Physical Activity

To measure physical activity, the German version of the WHO’s Global Physical Activity Questionnaire (GPAQ) was used [53]. The 16-item GPAQ differentiates physical activities of moderate and vigorous intensity and whether the activity was carried out during work, transport or recreational time. One item, for example, is “How much time do you spend doing moderate-intensity activities at work on a typical day?”. Based on the official Analysis Guide by the WHO [54], we calculated the total physical activity metabolic equivalents (METs, i.e., the ratio of the work metabolic rate to the resting metabolic rate) in minutes per week across the three areas of activity. More than 3000 min of physical activity per week can be classified as high and between 600 and 3000 min per week as moderate physical activity, whereas values less than 600 min per week are considered low values [55]. An international study confirmed the GPAQ’s good reliability (ranging from ρ = 0.67 to 0.81, *p* < 0.01) and acceptable concurrent validity (ranging from ρ = 0.45 to 0.65, *p* < 0.01) [56]. However, a more recent European study found lower values of concurrent validity (ρ = 0.11, *p* < 0.05 in general and ρ = 0.16, n.s. for the German version) [53].

#### 2.2.4. COVID-19 Pandemic-Related Variables

Worries and Degree of Worries

To assess concerns and worries regarding the ongoing COVID-19 pandemic among outpatient caregivers, the questionnaire for assessing general hospital staff’s worries towards the A/H1N1 pandemic developed by Goulia et al. (2010) [41] was adapted and translated into German. An initial dichotomous question was used to determine whether a respondent is worried about the COVID-19 pandemic (*yes* or *no*). If participants affirmed this question, they stated their most prevalent worries in a dichotomous format (dangerousness of pandemic, risk of infection for family and relatives, social isolation, consequences on functional abilities), indicating *yes* or *no*. Subsequently, their degree of worries was measured on a 9-point Likert scale (“How would you score your degree of worry?”), ranging from 1 = *I strongly disagree* to 9 = *I strongly agree*. The authors did not provide information on the scale’s reliability or validity [41]. The scale has already been used for assessment in the context of the COVID-19 pandemic in a previous study [30].

Perceived Sufficiency of Information

For the assessment of perceived sufficiency of information regarding the COVID-19 pandemic we adapted four items from another scale from the context of the swine flu pandemic [41] to the current COVID-19 pandemic context (e.g., “I believe that I have heard sufficient information about the symptoms of the coronavirus”) and translated them into German. The items were presented on a 9-point Likert scale (from 1 = *I strongly disagree* to 9 = *I strongly* *agree*). The original scale was sufficiently reliable (Cronbach’s α = 0.68 to 0.89) but not validated [41]. It has been adapted to the COVID-19 context before [30].

#### 2.2.5. Perceived Stress

Perceived stress was measured using the German version of the 10-item Perceived Stress Scale (PSS-10) [57,58]. The scale refers to stress experienced in the past month (e.g., “In the last month, how often have you felt nervous and stressed?”) and is rated on a five-point Likert scale (0 = *never*, 1 = *almost never*, 2 = *sometimes*, 3 = *fairly often*, 4 = *very often*). Since the PSS-10 was not developed for diagnostic purposes, there are no cut-off scores. Thus, higher scores indicate higher perceived stress. The German version of PSS-10 was validated (CFI = 0.96; TLI = 0.95; RMSEA = 0.07) and considered reliable (Cronbach’s α = 0.84) [57].

### 2.3. Statistical Methods

Initially, data of the 171 completed surveys were checked for plausibility, outliers and missing values. Subsequently, *n* = 16 cases were excluded from further data analysis due to missing or implausible values. Descriptive analysis of the data were performed with the remaining sample of *N* = 155 participants, including a correlation analysis for the score variables. In an exploratory approach, the distribution of the data was examined. Due to a lack of normal distribution, we decided to subdivide all of the scales into tertiles. This approach was chosen due to the mandatory three-level categorisation of the health literacy scale. Continuing this approach, the other scales were divided into tertiles based on their value distributions. This three-level categorisation was not only chosen based on the threefold categorisation of the health literacy scale, but also to avoid further loss of information in the sense of dichotomising the scales for binary logistic regression analyses. Finally, ordinal logistic regression analyses were used for testing the exploratory hypotheses. All statistical analyses were performed with IBM^®^ SPSS^®^ Statistics (version 25).

## 3. Results

### 3.1. Descriptive Results

The majority of participants (*N* = 155) were female (66.5%), aged between 35 and 39 years (19.4%), and were mobile by car working in outpatient care (70.3%). Individual participants whose work-related mobility did not fall into the proposed categories reported to work mobile by both, car and bike, work from the office or as care managers or working in day-care nursing as well. Work experience ranged from one to 33 years, with an average of 12.16 years (SD = 8.36 years). Similarly, participants’ job tenure with their current employers ranged from one to 30 years (M = 8.09 years, SD = 7.04 years). With regard to individual health-related characteristics, participants’ height ranged from 1.48 m to 1.90 m (M = 1.73 m, SD = 0.09 m), while their individual body weight ranged from 45 kg to 150 kg (M = 76.96 kg, SD = 16.49 kg). Resulting BMI according to WHO classification [43] and further details are provided in Table 1.

Regarding the main variables, participants showed an average health literacy score that is considered to be a sufficient level of health literacy [45]. More than half of the participants (*n* = 107 or 69%) showed a sufficient level of health literacy. Ten participants (6.5%) had an inadequate level of health literacy and 38 of the surveyed outpatient caregivers (24.5%) showed problematic levels of health literacy. The average MEDAS score indicates that participants only adhered to less than half of the 14 categories of the score. GPAQ values show an average of four and a half hours of physical activity per day during work and leisure time. Based on their MET-minutes, 84.5% (*n* = 131) of all participants were highly physically active, 13.5% (*n* = 21) were moderately physically active and only three participants (1.9%) were very little physically active. Perceived information sufficiency was rated with 7 out of 9 on average, whereas pandemic-related worries on average not only scored a lower mean, with 4 points on the 9-point scale, but also showed a smaller dispersion of values. Outpatient caregivers in our sample rated their perceived stress perception with an average of more than 16 out of a possible 40 points. As can be seen in the variable characteristics in Table 2, most of the scales used in this study showed acceptable to very good internal consistencies, except for the GPAQ.

Moreover, there was only one significant correlation among the main variables, between pandemic-related worries and perceived information sufficiency (Spearman’s ρ = −0.214, *p* < 0.001). Therefore, multicollinearity cannot be assumed as a precondition for ordinal regression analysis. Gender was significantly correlated with eating behaviour (Spearman’s ρ = 0.291, *p* < 0.001), pandemic-related worries (Spearman’s ρ = −0.196, *p* < 0.05) and perceived information sufficiency (Spearman’s ρ = 0.325, *p* < 0.001). Perceived information sufficiency was also correlated with age (Spearman’s ρ = −0.178, *p* < 0.05) and working full time (Spearman’s ρ = 0.174, *p* < 0.05). Further significant correlations were found between shift work and physical activity (Spearman’s ρ = 0.378, *p* < 0.001), working full time and pandemic-related worries (Spearman’s ρ = −0.160, *p* < 0.05), and among German mother tongue and perceived stress (Spearman’s ρ = 0.210, *p* < 0.001) and pandemic-related worries (Spearman’s ρ = 0.209, *p* < 0.001). Finally, educational level was significantly correlated with health literacy (Spearman’s ρ = 0.158, *p* < 0.05) as well as with physical activity (Spearman’s ρ = −0.339, *p* < 0.001). More details are provided in Table 3.

### 3.2. Main Results

Initially, we further examined the associations of health literacy with specific sociodemographic variables. We tested whether there were significant differences in health literacy with respect to different genders, age groups, educational levels or work experience. Therefore, Kruskal–Wallis tests for non-parametric data were conducted. The tests revealed an association of health literacy and educational level. Those participants who had a higher educational level also rated higher values of health literacy (χ^2^(3) = 11.418, *p* = 0.010). No such associations were found for health literacy and gender (χ^2^(2) = 0.236, *p* = 0.889), age (χ^2^(8) = 12.862, *p* = 0.117) and work experience (χ^2^(28) = 30.281, *p* = 0.350).

Testing our first assumption did not confirm the assumed association between health literacy and perceived information sufficiency with regard to the COVID-19 pandemic (χ^2^(2) = 1.748, *p* = 0.417). Our second assumption that outpatient caregivers’ health literacy would be associated with health behaviours could neither be supported. Health literacy as a predictor could neither significantly account for an amount of variance in eating behaviour (χ^2^(2) = 0.994, *p* = 0.608) nor for physical activity (χ^2^(2) = 2.085, *p* = 0.353). Moreover, the model for an association of outpatient caregivers’ health literacy with their pandemic-related worries was not significant (χ^2^(2) = 5.470, *p* = 0.065), as suggested in our third assumption. However, there was a significant negative effect for an inadequate level of health literacy and pandemic-related worries (*b* = −1.505, *SE* = 0.666, Wald χ^2^ = 5.110, *p* = 0.024), indicating 0.222 higher odds (and therefore decreasing probability) of worrying more due to the pandemic compared to participants with sufficient health literacy.

Moreover, our assumed association between outpatient caregivers’ pandemic-related worries and their physical activity (χ^2^(2) = 1.549, *p* = 0.461) was not significant either (Assumption 4b). In contrast, we found a significant association between the outpatient caregivers’ pandemic-related worries and their diet (χ^2^(2) = 7.831, *p* = 0.020), indicating that the probability for a higher MEDAS score (thus, a healthier diet) increased when outpatient caregivers showed a low (*b* = 0.935, *SE* = 0.378, Wald χ^2^ = 6.120, *p* = 0.013) or moderate (*b* = 0.845, *SE* = 0.366, Wald χ^2^ = 5.325, *p* = 0.021) degree of pandemic-related worries compared to highly worried participants (Assumption 4a). Thus, the odds of eating healthier were 2.546 (hardly worried) and 2.329 (moderately worried) times higher than for outpatient caregivers who were highly worried.

Our fifth assumption of an association between perceived information sufficiency and perceived stress could be confirmed as well (χ^2^(2) = 10.361, *p* = 0.006). Ordinal regression results indicated 3.194 times higher odds for higher levels of perceived stress when perceived information sufficiency was moderate compared to being well-informed (*b* = 1.161, *SE* = 0.371, Wald χ^2^ = 9.774, *p* = 0.002). Moreover, the sixth assumption that outpatient caregivers’ perceived sufficiency of information was associated with their pandemic-related worries could be supported based on ordinal regression results (χ^2^(2) = 15.675, *p* = 0.000). The probability for higher levels of pandemic-related worries was higher for outpatient caregivers who were hardly (*b* = 1.123, *SE* = 0.3803, Wald χ^2^ = 8.921, *p* = 0.003) or moderately well-informed (*b* = 1.445, *SE* = 0.380, Wald χ^2^ = 14.695, *p* < 0.001) about the COVID-19 pandemic. Thus, the odds of outpatient caregivers being more worried with regard to the COVID-19 pandemic were 3.073 times higher for those who were hardly and 4.243 times higher for those who were moderately well-informed about the COVID-19 pandemic compared to outpatient caregivers who were very well-informed.

Further results do not support our assumed relationship between outpatient caregivers’ pandemic-related worries and their level of perceived stress (χ^2^(2) = 3.291, *p* = 0.193) as supposed in Assumption 7. As direct associations were not found in the first place, the assumed moderations of pandemic-related worries between health literacy and health behaviours could only be partly supported as well. Ordinal regression results revealed no significant results for physical activity (χ^2^(7) = 5.794, *p* = 0.564) as in Assumption 8b. Yet, pandemic-related worries partly moderated the association between health literacy and eating behaviour (χ^2^(7) = 17.731, *p* = 0.013). There was a significant moderation when health literacy was problematic and outpatient caregivers were hardly worried about the pandemic (*b* = 1.629, *SE* = 0.630, Wald χ^2^ = 6.745, *p* = 0.009), with 5.096 times higher odds of showing a healthy eating behaviour than participants with adequate health literacy who were highly worried. Moreover, we found a significant moderation effect for adequate health literacy and a moderate degree of pandemic-related worries (*b* = 1.031, *SE* = 0.430, Wald χ^2^ = 5.856, *p* = 0.016), indicating 2.803 times higher odds of a healthy diet when participants were moderately worried about the pandemic. Assumption 8a could therefore be partially confirmed.

Lastly, pandemic-related worries also moderated the previously confirmed association of perceived information sufficiency and perceived stress among outpatient caregivers (χ^2^(8) = 16.036, *p* = 0.042) as supposed in Assumption 9. Thus, the odds of outpatient caregivers being more stressed were 4.040 times higher when they were moderately well-informed and highly worried (*b* = 1.396, *SE* = 0.640, Wald χ^2^ = 4.817, *p* = 0.028), indicating that having many worries concerning the COVID-19 pandemic led to more perceived stress among participants when they also perceived to be quite well-informed about the pandemic. Further details are provided in the Supplementary Materials.

## 4. Discussion

The current state of research provides little evidence considering outpatient caregivers’ health literacy and health-related outcomes, especially within the context of the COVID-19 pandemic. Our exploratory analyses contribute initial findings to the current state of research and thus provide a starting point for further research. Results from our sample did not show associations of health literacy and health behaviour, e.g., adherence to Mediterranean diet or physical activity or perceived information sufficiency regarding the COVID-19 pandemic. However, an association of educational levels and health literacy among outpatient caregivers was found. We were also able to show associations between perceived information sufficiency and pandemic-related worries and perceived stress, respectively. Moreover, great pandemic-related worries were found to amplify stress perception when perceived information sufficiency was moderate. COVID-19-related worries also partially moderated the association between health literacy and eating behaviour. However, they did not moderate the association of health literacy and physical activity.

Generally, our participants showed high levels of health literacy compared to the general German population [16] and another study among outpatient caregivers in Southern Germany [10]. The MEDAS score as a measurement for overall dietary quality revealed slightly lower values compared to the general Italian population during the COVID-19 lockdown [59], although a generally higher adherence to the Mediterranean diet could be expected in their sample due to the geographical location and cultural aspects. In agreement with these results, a mixed-methods study from the COVID-19 context, outpatient caregivers in Germany showed unchanged or slightly deteriorating eating behaviour [40]. Similarly, physical activity had not decreased due to the COVID-19 pandemic according to a German panel survey [60]. However, a systematic review reports decreased physical activity during the COVID-19 pandemic lockdown [61], which is also consistent with the findings of the mixed-methods study among outpatient caregivers [40]. Although there are no cut-off values for the PSS-10 since it is not a diagnostic instrument [57], the average score may be considered a moderate stress perception when compared to mean values of the general US population [58,62]. However, the PSS-10-mean values among our sample of outpatient caregivers are comparable to those of other healthcare professionals during the COVID-19 pandemic [63,64]. The average pandemic-related worries of outpatient caregivers are also comparable to the extent of worries among nursing staff in the original study of the scale, whereas perceived information sufficiency was rated higher in our sample [41].

### 4.1. Health Literacy with Regard to Gender, Age, Work Experience and Educational Level

With regard to different sociodemographic variables, we found an association of educational level and health literacy among outpatient caregivers in our sample. In contrast, gender, age, and work experience were not associated with health literacy. These results are supported by a study among the general German population (*N* = 4952), where a similar association of sociodemographic data with health literacy was found. Gender and age were not associated with health literacy, but educational attainment was [16]. There are some studies which report lower education and health literacy levels in different contexts (cf. [65,66,67]).

### 4.2. Health Literacy and Pandemic-Related Perceived Information Sufficiency

In our sample of outpatient caregivers, health literacy was not associated with perceived information sufficiency regarding the COVID-19 pandemic. Surveys conducted before the pandemic indicate a lack of knowledge among caregivers which could even reflected in outpatient care due to the COVID-19 pandemic [68]. Insufficient knowledge about H1N1 influenza was shown among healthcare workers in spite of relevant training [69]. Study results from the inpatient care setting show that perceived information sufficiency among nurses was moderately high regarding certain aspects of the A/H1N1 influenza. The overall information about the A/H1N1 influenza was reported to be perceived as clear (mean: 7.0 ± SD: 1.6, median: 7.4 on the same 9-point scale used in the present study) [41], which is marginally lower than in our sample regarding the COVID-19 pandemic. Generally, health literacy levels were comparatively high in our sample which may reduce the perceived sufficiency of additional COVID-19-related information among the surveyed outpatient caregivers.

### 4.3. Health Literacy and Changing Health Behaviours before and during the COVID-19 Pandemic

In the present study, exploratory analyses did not reveal significant relations between health literacy and health behaviours among outpatient caregivers. However, other research results show that health literacy can result in changed health behaviour. Higher health literacy levels are often associated with healthier behaviour in the general population [17,70]. Among 1797 trainees, including geriatric nurses (*n* = 138), decreasing health literacy was associated with showing less health-promoting behaviours with respect to aspects such as diet and physical exercise [71]. With regard to the current COVID-19 pandemic, recent literature discusses the protective potential of a balanced diet and sufficient nutritional status against such viral infections [72,73,74]. However, the ongoing pandemic can also represent a stressor, leading to changes in health behaviours [59,75]. While the findings of a pre-COVID-19 Polish study among nurses (*n* = 1080) indicate that lower psychophysical mood was associated with a greater tendency to develop improper eating habits in a non-pandemic context [76], findings by a recent mixed methods-study among outpatient caregivers from Germany revealed unchanged or deteriorating eating behaviour for the majority of participants, as well as less physical activity during the COVID-19 pandemic [40].

Despite the insignificant association of health literacy and health behaviours in the present study, we found a significant association between pandemic-related worries and a healthy diet, but not physical activity. Similarly, a low and moderate degree of pandemic-related worries moderated the association between health literacy and diet, but not physical activity. According to the Health Belief Model, health behaviour can be reflected in the uptake of preventive measures. The display of health behaviour depends on the perceived threat (i.e., of the coronavirus) which is influenced by the perceived susceptibility and seriousness of the disease, in this case COVID-19, demographic variables and cues to action [77]. Since outpatient caregivers are working in the field, in a mobile setting, and visit numerous patients who are especially vulnerable to COVID-19 on a daily basis, they will probably perceive their susceptibility as high. Comparatively high levels of health literacy among our participants could indicate they also understand the seriousness of an infection. Looking at our results it can be hypothesised that a certain level (i.e., problematic or sufficient) of health literacy would be necessary to appraise the circumstances as serious and, in combination with being little or moderately worried, may lead to a change in health behaviour (i.e., a healthier diet). On the contrary, being highly worried could possibly paralyse them and discourage behavioural changes to healthier ones. However, the question as to why there is no significant effect with regard to physical activity remains unanswered. This is possibly related to the high level of job-related physical activity or restrictions such as the closure of gyms during the lockdown, as suggested by German outpatient caregivers in another study [40].

### 4.4. Health Literacy and Pandemic-Related Worries

In our sample, we found only partially significant results confirming an association between inadequate health literacy and greater pandemic-related worries. Similarly, higher degrees of worry in the context of lower health literacy levels are already known from the pre-pandemic context among cancer patients, students, or patients with coronary heart diseases [7,9,78]. Whereas health literacy and worries have often been researched in cancer patients to date (e.g., [79,80,81,82]), there is a lack of research among outpatient caregivers. However, health literacy becomes relevant for decreasing COVID-19 pandemic-related worries, as it can be a protective factor regarding fears caused by pandemic situations [28,83,84]. 

### 4.5. Perceived Information Sufficiency and Stress Perception

Participants were more likely to perceive more stress when they perceived themselves to be quite (moderately) sufficiently informed about the COVID-19 pandemic. Interaction effects indicate that great worries cause more stress when outpatient caregivers were moderately well-informed. Similarly, in a study among German caregiving relatives who felt they were sufficiently informed about the ongoing pandemic situation, they were emotionally stressed and exhausted [36]. Perception of stress due to an unfamiliar work environment is also known among intensive care unit nurses [85], frontline nurses [86], and ambulance nurses [37]. Uncertainty about the COVID-19 pandemic was also named as the most frequently cited stressor among doctors and nurses from a German hospital. This led to increased stress perception, fatigue and depressive symptoms, even more among nurses than among doctors [87]. Other international studies also describe adverse health effects among healthcare workers resulting from uncertainty and misinformation due to the COVID-19 pandemic [88,89,90,91]. Preti et al. (2020) therefore recommended the provision of protective materials in combination with frequent information [92].

### 4.6. Perceived Information Sufficiency and Pandemic-Related Worries

Our results support the assumption that outpatient caregivers’ perceived sufficiency of information is associated with their pandemic-related worries. Rather contradictory results can be found with regard to concerns about the COVID-19 pandemic in connection with information status [84,93]. Although we found comparatively high levels of perceived information sufficiency in our sample [41], globally, insufficient knowledge about COVID-19 was found among stationary healthcare workers [34,94]. Italian and Japanese healthcare workers were constantly worried because of the COVID-19 pandemic and rated their risk of getting infected as very high [94,95], which was also already shown among German outpatient caregivers [39]. Moreover, in another pandemic context, insufficient knowledge about H1N1 influenza was shown among healthcare workers despite relevant training [69]. However, during the MERS-CoV pandemic, healthcare workers showed a more positive attitude towards MERS-CoV with increasing knowledge [96]. In addition, a study of 2007 Taiwanese people, including healthcare workers (*N* = 647, of which *n* = 123 nurses), showed how information sources, such as internet and traditional media, fellow human beings (family, co-workers, friends, etc.) can have an impact on respondents’ pandemic-related worries. In particular, internet media triggered high levels of public worry. Information obtained from academic courses (formal courses led by experts, personal or online), in contrast, resulted in lower levels of worry [97].

### 4.7. Pandemic-Related Worries and Perceived Stress

Although great pandemic-related worries amplified the association between moderate information sufficiency and stress perception, we did not find pandemic-related worries to be directly associated with perceived stress in our presented study. This result is rather striking, since several study results highlight that nurses worried about their own infection or the risk of infecting others (relatives, patients) in the care setting, ultimately resulting in higher perceived stress levels [38,85,86,98,99,100,101,102]. Unfortunately, data from our survey do not offer more in-depth explanations on the emergence of worries and perceived stress. However, other studies point to explanations: for example, a shortage of personal protective equipment (PPE) was a main reason for outpatient caregivers being worried at the beginning of the outbreak of the COVID-19 pandemic, which eventually caused a high stress perception [15,39,103]. This is also known from other research from the inpatient care setting [86,104,105], from the partially inpatient and partially outpatient care sector [101,106], and among healthcare workers [107]. Regarding the long survey period, the shortage had possibly already been overcome by the time of participation of many respondents.

Yet, from the outpatient care setting, study results indicate that German outpatient caregivers also exhibited constant worry and anxiety due to the COVID-19 pandemic and reported increased stress perception on a daily basis [39]. Increased stress perception during the COVID-19 pandemic was also reported by Mojtahedzadeh, Neumann et al. (2021) in their mixed-methods research among outpatient caregivers from Germany [40]. However, despite lacking comparative values of our participants before the COVID-19 pandemic, stress perception among our sample seemed rather moderate.

### 4.8. Strengths and Limitations

A strength of our study is that we were able to recruit outpatient caregivers from different care services of different city districts from Northern Germany despite the possibly limited time due to high work density, time pressure and shift work [108], and increased job demands in outpatient care during the COVID-19 pandemic in Germany [39]. Furthermore, our study sample of 155 participants had different sociodemographic characteristics, e.g., it included different ages, work experience lengths, and types of employment. Another strength of our survey is the use of validated instruments. By using an online questionnaire, we were able to ensure that no data went missing. Overall, we were able to gain initial insights on this so-far-unexplored topic, and we have therefore contributed to closing the above identified research gap.

However, there are some limitations that should be noted. Our sample size of 155 outpatient caregivers comprises 0.04% of our target population (outpatient caregivers working in German outpatient care services, *N* = 421,550 cf. [11]). Furthermore, the participation rate was low, and the drop-out rate was high. Therefore, the extent of representativeness is limited. The lack of research including health literacy, health behaviour, pandemic-related worries, perceived information sufficiency and stress has prompted us to refer to literature from other, more general contexts and occupational groups, such healthcare workers, nurses, or geriatric care. This literature, however, can only be considered carefully in relation to our findings.

To generate more study participants, a long survey period over several pandemic waves was necessary, which, however, may also have resulted in bias, as recent research findings suggest that health behaviour changes over time [109]. Moreover, different waves of the COVID-19 pandemic within the data collection period might have not only changed health behaviours, but also influenced variables such as pandemic-related worries, perceived information sufficiency, or perceived stress. In addition, due to the self-assessment of the participants with respect to the survey, it must be mentioned that social desirability bias and possible errors of judgement cannot be ruled out [110]. As outpatient caregivers are responsible for patients in their work [12], they may be required to have a certain level of physical fitness and mental health. Surveyed outpatient caregivers might therefore have given answers that seem socially acceptable to them [111,112]. Especially in questions about eating behaviour and physical activity, social desirability seems to carry a risk of bias [113,114,115,116,117,118].

Moreover, nurses from the inpatient care have also been observed to have the highest health literacy awareness among hospital employee groups [119], which could have a self-selection bias effect on our participants as well. Moreover, the healthy worker effect cannot be ruled out in our sample of outpatient caregivers [120]. Because there is no pre–post comparison, the data may moreover be retrospectively biased, and the validity of the results may be limited. Furthermore, the measurement instruments are subject to certain limitations. Potential bias can arise due to differences in other populations’ lifestyles and diets diet in a non-Mediterranean context by using the MEDAS [121]. However, the MEDAS was considered as an appropriate tool for our survey as it has been validated for the German population regarding dietary quality in international guidelines [47,48]. Although the reliability of the GPAQ was assessed as “good” to “very good”, the concurrent validity requires further investigation [122]. Nevertheless, the GPAQ is widely used in Germany and has already been validated [53]. However, we noticed some implausible data concerning the GPAQ among the present sample, which we consequently excluded from data analysis. Therefore, we assume that some participants might not have understood the logic of the scale and its structure properly and might have needed some more explanation. This, again, relates to the quantitative nature of our study. 

### 4.9. Implications for Further Research and Practice

#### 4.9.1. Implications for Research

As indicated above, further research studies with larger sample sizes are needed in the future to obtain more representative results for the outpatient care setting, since outpatient caregivers are a special occupational group due to their specific mobile work environment and higher job demands because of the pandemic [39]. Moreover, longitudinal studies would provide further valuable insights to be able to record possible changes during the COVID-19 pandemic at different points in time [110]. Although there are some reflections on the extent to which health literacy is stable over time [123,124], it seems plausible that an individual’s level of health literacy mainly changes due to educational, aging or pathological processes [123,124,125,126]. For future studies, it could therefore be interesting to conduct surveys with outpatient caregivers who have experienced the COVID-19 pandemic for more than one year and a half now, and how their health literacy levels, perceived information sufficiency and pandemic-related worries might have changed over time. In the meantime, various vaccines are available to protect against COVID-19, and outpatient caregivers were already among the prioritised groups in Germany [127,128]. Recently published study results by Kozak and Nienhaus (2021) indicate that there is already a high willingness to vaccinate among geriatric care nurses, yet there are some who are still hesitant about the vaccination [129]. Hence, it would be of research interest to examine the status quo of vaccinated outpatient caregivers (vaccinated vs. non-vaccinated) and their health literacy or pandemic-related worries during the COVID 19 pandemic. Finally, with larger sample sizes, it could be a future interest in research to consider, e.g., different ages and gender distribution. By applying such mixed-methods studies, quantitative survey results could be further explained by qualitative research results [130]. Finally, it must be emphasised that the HLS-EU-Q16, which we used to measure health literacy, has proven to be a valid instrument for the general population [44,46,131]. For further research, however, it would be desirable to develop a care-specific instrument and to use it for further studies, especially since we found above-average levels of health literacy in our sample [10,16]. After more research has been carried out, specific interventions within the framework of workplace health promotion and occupational health and safety could be developed and implemented [132]. 

#### 4.9.2. Implications for Practice

Implications for practice can be divided into behavioural prevention measures (e.g., improving coping competencies) and structural prevention measures (adjusting work organisation and environment) [133,134,135]. Outpatient caregivers should be educated about a healthy diet and sufficient physical activity (cf. [134]), as a higher health literacy could imply a better health behaviour by putting knowledge into practice [13]. This is especially important, since we found rather high levels of health literacy among participants, but average or low values for health behaviour (i.e., eating behaviour and physical activity). In addition, personal resources should be strengthened in general, as this could decrease perceptions of stress [134,136]. Improving outpatient caregivers’ resilience in general could result in a reduced stress perception and decrease symptoms of anxiety [134,137,138]. Although behavioural interventions are of great importance, it seems to be more difficult for the target population to adapt the measures in practice [139]. Therefore, preventions on the structural level need to be targeted with greater focus [140].

On the structural level, employers in the outpatient care should always provide sufficient offers of information in relation to the COVID-19 pandemic, which can lead to a decrease in stress perception and worries in times of a pandemic [41]. Overall, workplace health promotion offers, e.g., interventions to promote resilience and information sessions on health-related topics, should be offered by employers in outpatient care. With regard to social distancing regulations [141], health promotion programmes could also be offered via corresponding online tools to ensure health-promoting behaviour (e.g., [142]). Furthermore, the positive effects of social support [143], team spirit, communication, social exchange between colleagues and superiors should be ensured despite of the ongoing COVID-19 pandemic to inhibit feelings of isolation [144,145]. The specific development of structural interventions, however, remains important [132,146], particularly because German outpatient caregivers are constantly getting older [11]. All in all, improving the health literacy of outpatient caregivers could not only encourage the conduct of more health-promoting behaviours, but also improve their general health, motivation and productivity [13,108]. This needs to be given more attention during the COVID-19 pandemic [15,39]. Finally, increased health literacy can also lead to improved communication with patients in the long term [147], as patients have a greater need to talk to outpatient caregivers during the pandemic [39]. Research findings indicate that health literacy can be trained among healthcare professionals [148]. Although outpatient caregivers in the present sample showed high values on the health literacy scale, specific training on translating their knowledge into actions could improve health behaviour. In addition, it would be advisable to provide outpatient caregivers with sufficient PPE at all times in order to avoid anxiety and other negative strain reactions, such as stress perception [15,39].

## 5. Conclusions

This cross-sectional study focused on the health literacy of outpatient caregivers during the COVID-19 pandemic, a mostly unexplored field [10]. Our findings indicate a high differentiation of insights into individual levels of health literacy in association with perceived information sufficiency, pandemic-related worries, and stress perception of outpatient caregivers during the COVID-19 pandemic. Health behaviours (physical activity, diet) were not related to levels of health literacy among our participants. Perceived sufficiency of information was associated with pandemic-related worries and stress perception. Moreover, pandemic-related worries moderated the relationship between perceived information sufficiency and stress perception. It could be helpful to ensure access to information to provide reliable and appropriate knowledge about COVID-19 and to promote outpatient caregivers’ health literacy and strengthen their personal resources. Possible negative strain reactions, such as stress, resulting from false or insufficient information and worries could be avoided in this way in the future and thereby promote outpatient caregivers’ health [13,149]. These aspects should be further considered when planning a better work environment in outpatient care services during the COVID-19 pandemic.

## Figures and Tables

**Table 1:** Sociodemographic characteristics of participants (*N* = 155).

Title 1	*n*	%
Gender		
Male	50	32.3
Female	103	66.5
Diverse	2	1.3
Age		
≤24 years	7	4.5
25–29 years	10	6.5
30–34 years	18	11.6
35–39 years	30	19.4
40–44 years	18	11.6
45–49 years	17	11.0
50–54 years	22	14.2
55–59 years	21	13.5
≥60 years	12	7.7
Work area		
Outpatient care (mobile by car)	109	70.3
Outpatient care (mobile by bike or foot)	24	15.5
Outpatient care in retirement or nursing home (no mobility)	5	3.2
Intensive care (no mobility required)	3	1.9
Other	14	9.0
Type of employment		
Temporary contract	15	9.7
Permanent contract	140	90.3
Part-time	80	51.6
Full-time	75	48.4
Shift work		
Yes	83	53.5
No	72	46.5
Managerial responsibility		
Yes	54	34.8
No	101	65.2
Highest educational level		
General secondary school	14	9.0
Intermediate secondary school	80	51.6
Specialised grammar school	19	12.3
Grammar School	42	27.1
Family status		
Single	52	33.5
Married	86	55.5
Registered civil partnership	7	4.5
Divorced	9	5.8
Widowed	1	0.6
Children		
No children	85	54.8
1 child	54	34.8
2 children	12	7.7
3 children	4	2.6
Living in Germany		
Since birth	132	85.2
Later in life	23	14.8
German mother tongue		
Yes	137	88.4
No	18	11.6
BMI		
Underweight ≤ 18.5	2	1.3
Normal weight 18.5 ≤ 24.9	93	60.0
Overweight/pre-obesity 25 ≤ 29.9	34	21.9
Obesity (classes I, II, III) ≥ 30	26	16.8

**Table 2:** Variable characteristics and internal consistencies of scales.

Scale	Range	Min	Max	Mean	Median	SD	α
Health literacy	0–16	0	16	13.290	14.000	2.996	0.899
Eating behaviour	0–14	1	11	6.323	6.000	2.239	0.508
Physical activity	-	240	38,880.000	9872.981	8160.000	7933.685	0.323
Perceived information sufficiency	1–9	1.500	9.000	7.097	7.750	2.028	0.919
Pandemic-related worries	1–9	1.167	7.167	4.369	4.333	1.456	0.682
Perceived stress	0–40	7	27	16.529	17.000	4.237	0.761

Note. SD indicates standard deviations and α (Cronbach’s alpha) was used as a reliability measure for internal consistency. Physical activity was transformed into metabolic equivalents for data analyses. Cronbach’s α refers to the GPAQ’s physical activity scales excluding sedentary behaviour.

**Table 3:** Correlation between variables and sociodemographic characteristics (Spearman’s ρ, *N* = 155).

Variable	Health Literacy	Eating Behaviour	Physical Activity	Perceived Information Sufficiency	Pandemic-Related Worries	Perceived Stress
Health literacy	-					
Eating behaviour	0.081	-				
Physical activity	−0.018	−0.004	-			
Perceived information sufficiency	−0.054	−0.045	0.086	-		
Pandemic-related worries	0.096	−0.130	0.080	−0.214 **	-	
Perceived stress	−0.058	0.060	0.103	−0.114	0.120	-
Gender	−0.006	0.291 **	0.117	0.325 **	−0.196 *	0.029
Age	0.145	0.021	0.031	−0.178 *	0.084	−0.056
Work experience	0.042	0.038	0.010	0.035	0.027	−0.008
Shift work	−0.061	0.028	0.378 **	−0.084	0.135	0.039
Full-time job	−0.048	0.150	−0.070	0.174 *	−0.160 *	0.036
German mother tongue	−0.088	−0.045	0.083	0.144	0.209 **	0.210 **
Educational level	0.158 *	0.110	−0.339 **	−0.069	−0.077	−0.118

Note. * *p* < 0.05, ** *p* < 0.001.

## Data Availability

The datasets generated and/or analysed during the current study are not publicly available due to German data protection regulations, but are available from the corresponding author on reasonable request.

## Supplementary Materials

The following are available online at https://www.mdpi.com/article/10.3390/ijerph182211743/s1, Table S1: Results of associations with perceived information sufficiency from ordinal logistic regression (*N* = 155), Table S2: Results of associations with eating behaviour from ordinal logistic regression (*N* = 155), Table S3: Results of associations with physical activity from ordinal logistic regression (*N* = 155), Table S4: Results of associations with pandemic-related worries from ordinal logistic regression (*N* = 155), Table S5: Results of associations with perceived stress from ordinal logistic regression (*N* = 155).

## Abbreviations

BMI	Body Mass Index
COVID-19	Coronavirus Disease 2019
GPAQ	Global Physical Activity Questionnaire
HLS-EU-Q16	European Health Literacy Survey Questionnaire
MEDAS	Mediterranean Diet Adherence Screener
MET	Metabolic Equivalent of Task
MERS	Middle East Respiratory Syndrome
PPE	Personal Protective Equipment
PSS-10	Perceived Stress Scale (10-item version)
Ref.	Reference Category
SARS	Severe Acute Respiratory Syndrome
WHO	World Health Organization
