# Supplementary material for: The Role of Health Literacy among Outpatient Caregivers during the COVID-19 Pandemic
Source: Int J Environ Res Public Health. 2021 Nov 9;18(22):11743. doi: 10.3390/ijerph182211743 (PMC8624592; doi:10.3390/ijerph182211743)
Supplement: Supplementary file 1 [file ijerph-18-11743-s001.zip › ijerph-1392006-supplementary.pdf]

Table S1. Results of associations with perceived information sufficiency from ordinal logistic regression ($N = 155$).

Variables	OR	95% CI Lower Bound	95% CI Upper Bound
Inadequate health literacy	1.043	0.317	3.431
Problematic health literacy	1.579	0.795	3.139
Sufficient health literacy (Ref.)	1.000	.	.

Note. CI = Confidence interval. Ref. = Reference category.

Table S2. Results of associations with eating behaviour from ordinal logistic regression ($N = 155$).

Variables	OR	95% CI Lower Bound	95% CI Upper Bound
Inadequate health literacy	0.553	0.159	1.920
Problematic health literacy	1.111	0.561	2.199
Sufficient health literacy (Ref.)	1.000	.	.
Hardly worried	**2.546**	**1.214**	**5.339**
Moderately worried	**2.329**	**1.136**	**4.776**
Highly worried (Ref.)	1.000	.	.
Inadequate health literacy * hardly worried	1.972	0.446	8.718
Inadequate health literacy * moderately worried [1]	-	-	-
Inadequate health literacy * highly worried	0.000	0.000	0.000
Problematic health literacy * hardly worried	**5.096**	**1.491**	**17.420**
Problematic health literacy * moderately worried	1.052	0.342	3.238
Problematic health literacy * highly worried	1.027	0.276	3.822
Sufficient health literacy * hardly worried	1.700	0.689	4.195
Sufficient health literacy * moderately worried	**2.803**	**1.216**	**6.460**
Sufficient health literacy * highly worried (Ref.)	1.000	.	.

Note. [1] No values in this cell. CI = Confidence interval. Ref. = Reference category. Significant values ($p < 0.05$) in bold type.

Table S3. Results of associations with physical activity from ordinal logistic regression ($N = 155$).

Variables	OR	95% CI Lower Bound	95% CI Upper Bound
Inadequate health literacy	2.037	0.599	6.924
Problematic health literacy	0.781	0.395	1.546
Sufficient health literacy (Ref.)	1.000	.	.
Hardly worried	0.882	0.430	1.810
Moderately worried	0.647	0.321	1.303
Highly worried (Ref.)	1.000	.	.
Inadequate health literacy * hardly worried	1.413	0.320	6.232
Inadequate health literacy * moderately worried [1]	-	-	-

Inadequate health literacy * highly worried	4.847	0.399	58.894
Problematic health literacy * hardly worried	0.578	0.180	1.852
Problematic health literacy * moderately worried	0.612	0.203	1.842
Problematic health literacy * highly worried	1.560	0.432	5.637
Sufficient health literacy * hardly worried	1.256	0.516	3.056
Sufficient health literacy * moderately worried	0.833	0.370	1.875
Sufficient health literacy * highly worried (Ref.)	1.000	.	.

Note. [1] No values in this cell. CI = Confidence interval. Ref. = Reference category.

Table S4. Results of associations with pandemic-related worries from ordinal logistic regression (*N* = 155).

Variables	OR	95% CI Lower Bound	95% CI Upper Bound
Inadequate health literacy	**0.222**	**0.060**	**0.819**
Problematic health literacy	0.649	0.328	1.286
Sufficient health literacy	1.000	.	.
Hardly informed	**3.073**	**1.471**	**6.421**
Moderately informed	**4.243**	**2.027**	**8.884**
Well-informed (Ref.)	1.000	.	.

Note. CI = Confidence interval. Ref. = Reference category. Significant values ($p < 0.05$) in bold type.

Table S5. Results of associations with perceived stress from ordinal logistic regression (*N* = 155).

Variables	OR	95% CI Lower Bound	95% CI Upper Bound
Hardly worried	0.568	0.275	1.174
Moderately worried	0.570	0.282	1.152
Highly worried (Ref.)	1.000	.	.
Hardly informed	2.018	0.974	4.182
Moderately informed	**3.194**	**1.542**	**6.614**
Well-informed (Ref.)	1.000	.	.
Hardly informed * hardly worried	3.751	0.736	19.117
Hardly informed * moderately worried	1.114	0.332	3.739
Hardly informed * highly worried	3.751	0.959	14.670
Moderately informed * hardly worried	2.747	0.583	12.948
Moderately informed * moderately worried	2.640	0.745	9.353
Moderately informed * highly worried	**4.040**	**1.161**	**14.055**
Well-informed * hardly worried	1.000	0.308	3.251

Well-informed * moderately worried	0.973	0.188	5.036
Well-informed * highly worried (Ref.)	1.000	.	.

Note. CI = Confidence interval. Ref. = Reference category. Significant values ($p < 0.05$) in bold type.
